# Identification of an Exosomal miRNA Signature in Newly Diagnosed Essential Hypertensive Adults

**DOI:** 10.3389/bjbs.2025.14780

**Published:** 2025-11-21

**Authors:** Paulina Pei Suu Tan, William M. Chilian, Yook Chin Chia, Shamsul Mohd Zain, Hooi Min Lim, Navin Kumar Devaraj, Siew Mooi Ching, Teck Yew Low, Nur Afrina Muhamad Hendri, Tg Rogayah Tg Abd Rashid, Yong Ling Sou, Yuh-Fen Pung

**Affiliations:** 1 Division of Biomedical Science, University of Nottingham Malaysia, Semenyih, Selangor, Malaysia; 2 Integrative Medical Sciences, Northeast Ohio Medical University, Rootstown, OH, United States; 3 Sir Jeffrey Cheah Sunway Medical School, Faculty of Medical and Life Sciences, Sunway University, Sunway University, Sunway, Malaysia; 4 Department of Primary Care Medicine, Faculty of Medicine, Universiti Malaya, Kuala Lumpur, Malaysia; 5 Department of Pharmacology, Universiti Malaya, Kuala Lumpur, Malaysia; 6 Department of Family Medicine, Faculty of Medicine and Health Sciences, Universiti Putra Malaysia, Serdang, Selangor, Malaysia; 7 Electron Microscopy Unit, Institute for Medical Research, Ministry of Health Malaysia, Setia Alam, Selangor, Malaysia

**Keywords:** cardiovascular disease, small extracellular vesicles, high blood pressure, precision medicine, biomarker discovery

## Abstract

**Background:**

Hypertension is a major risk factor for cardiovascular diseases and premature death worldwide. Less than half of adults with hypertension are not properly diagnosed and treated indicating a need for better diagnostic and treatment strategies. Exosomal miRNAs have been implicated in hypertension development and show potential as non-invasive disease biomarkers. Therefore, this study aimed to investigate potential exosomal miRNA biomarkers of hypertension to enhance early detection.

**Methods:**

Plasma exosomes from newly identified, stage I essential hypertensive adults and their controls were isolated and characterised. The miRNA profiles were compared using small RNA sequencing, then validated with quantitative PCR (qPCR). Enriched pathways and gene ontologies of predicted miRNA targets were compared against systemically dysregulated pathways to validate its biological function.

**Results:**

Hypertensives showed preferential release of exosomes larger than 150 and significantly reduced expression of exosomal CD9. After qPCR validation, a unique hypertensive exosomal miRNA profile consisting of three downregulated and one upregulated miRNA was identified. The combination of this miRNA signature (hsa-miR-184, hsa-miR-432-5p, hsa-miR-1-3p, and hsa-miR-1246) with BMI demonstrated the highest diagnostic value. Predicted target pathways of the miRNA signature and systemically dysregulated proteomics pathways highlighted the convergence of aberrant metabolic pathways in the development of hypertension.

**Conclusion:**

This study identified a unique hypertensive exosomal miRNA profile when used combination with BMI. The miRNA signature provided insights into the mechanisms involved in the early stages of hypertension and offers leads for further validation in biomarker discovery to alleviate the burden of cardiovascular diseases.

## Introduction

According to a World Health Organization (WHO) report in 2019, an estimated 1.4 billion people worldwide suffer from hypertension [1, 2]. In Malaysia, the National Health and Morbidity Survey 2019 (NHMS 2019) reported that 30.0% of the population were hypertensive. The prevalence of hypertension has not improved since 2011 based on previous NHMS reports. A secondary analysis of NHMS 2015 showed that including pre-hypertensive patients, almost 70% of Malaysians are at risk of elevated high blood pressure, indicating a large number of individuals that may develop cardiovascular disease in the future if left uncontrolled [3].

The diagnosis of hypertension requires multiple visits over several weeks to confirm consistent elevations in clinic blood pressure readings [4]. However, conventional blood pressure measurements may not effectively identify early cases of hypertension, particularly in asymptomatic individuals with primary or essential hypertension, which constitutes 95% of all hypertensive patients [5]. In the absence of regular blood pressure monitoring, these cases may go undetected, earning hypertension its reputation as a “silent killer.” The high statistic (47%) of hypertensive unawareness is alarming as hypertension significantly increases the risk of cardiovascular disease such as ischemic heart disease and stroke, which has been the leading cause of death in Malaysia for over two decades [6, 7]. Mortality statistics showed that efforts taken to improve the diagnosis of hypertension such as increased community blood pressure screenings and blood pressure management using conventional blood pressure measurement methods, were not effective [8, 9]. Therefore, there is an avenue for a more precise diagnostic marker to improve the detection of early hypertension.

MicroRNAs (miRNAs) are small regulatory molecules that are involved in post-transcriptional modifications and transported in extracellular carriers such as exosomes [10–14]. MiRNAs are considered master regulators of gene expression and can regulate virtually every cellular process, including development, proliferation, migration, survival, metabolism, homeostasis, and regeneration [15]. Computational analysis estimated that more than 60% of human protein coding genes are targeted by at least one miRNA. Therefore, aberrant miRNA expression can have detrimental effects on signalling pathways, leading to development of various diseases, including hypertension [16].

The emerging role of exosomal miRNA in hypertension pathophysiology presents promising targets for diagnostics and therapeutics. Studies have shown that miRNA can regulate components of the renin angiotensin system, as well as multiple processes that contribute to vascular remodelling and endothelial dysfunction [17–19]. Multiple mechanisms supporting the active and selective loading of miRNA into exosomes indicates its physiological significance and potential as biomarkers [20–26].

There is a potential for the use of exosomal miRNAs for early detection of hypertension. However, fundamental research is needed, particularly in Southeast Asian populations such as Malaysia to identify the association of exosomal miRNAs and hypertension in this population for the development of predictive biomarkers. This study aimed to compare the exosomal miRNA profile of newly diagnosed hypertensives and predict its functional significance against systemically dysregulated pathways to identify potential biomarkers for early detection of hypertension.

## Methods

### Ethics Approval, Recruitment Sites and Study Design

This study was designed as a case-control study to identify potential exosomal miRNA biomarkers associated with hypertension. The study protocol was approved by institutional ethics review board in University Malaya Medical Centre (UMMC) (MRECID.NO: 202031-8331), Universiti Putra Malaysia (UPM) (JKEUPM-2020-128), and the University of Nottingham Malaysia (YFP100120). The study was carried out in accordance with the principles outlined in the ethical guidelines of the 1975 Declaration of Helsinki and all participants signed a consent form prior to participation.

Participants were recruited from University Malaya (UMMC) and Hospital Pengajar Universiti Putra Malaysia (HPUPM) from April 2022 to February 2023. Six hypertensive patients and their matched controls were recruited for miRNA discovery using next-generation sequencing (NGS) using quota sampling to ensure representation across the main ethnic groups in Malaysia. Another 18 hypertensive patients and 17 controls were recruited by random sampling for miRNA validation using RT-qPCR. Three hypertensive patients and their matched controls were randomly selected from the validation cohort for SOMAscan analysis (proteomics) to identify systemically dysregulated pathways in hypertension to support the predicted enriched pathways of differentially expressed miRNA targets.

### Sample Size Calculation

Sample size was estimated using the G* Power version 3.1.9.4 software adopting an *a priori* two-tailed *t*-test of with the following assumptions: effect size = 1.414, α = 0.05 and power (1 – β) = 0.95. As hypertension is linked to vascular aging, the effect size was computed based on the differences of mean of exosomal miRNA expression and standard deviation between young and senescent human VSMCs [27]. Following this, the minimum sample size was estimated to be 30 subjects, with 15 subjects per cohort. Considering 20% dropout rate, a total of 36 subjects and 18 subjects per group were required [28].

### Participant Recruitment and Eligibility

Inclusion criteria were hypertensive patients between 30 and 55 years old, who were newly diagnosed stage 1 essential hypertensives (SBP = 140–159 mmHg and/or DBP = 90–99 mmHg) [29] with no prior history of hypertension and had not received any treatment. Any patients who were hypertensives with secondary causes, morbidly obese (body mass index, BMI ≥40.0) or patients with cancer, liver, kidney, heart disease, stroke, or psychiatric disorders, diabetes, and pregnant or lactating individuals were excluded. The controls were age-, gender-, and ethnicity-matched adults without hypertension (SBP <129 mmHg and/or DBP <84 mmHg) and satisfied the exclusion criteria.

### Blood Pressure and Anthropometric Measurements

Reported blood pressure was measured using the Rossmax Automatic Blood Pressure Monitor (X1) (Rossmax, Taipei, Taiwan), which was approved by STRIDE BP. Subjects rested in a sitting position for at least 5 min prior to blood pressure measurements. Blood pressure was measured twice with a one to 2 min interval between each reading and the average of the two measurements was taken. Height and waist circumference were measured to the nearest 0.5 cm. The weight was measured to the nearest 0.1 kg. Abdominal obesity was defined as waist circumference ≥90 cm for men, and ≥80 cm for women, according to the Malaysian Clinical Practice Guidelines on Management for Obesity [30].

### Blood Sampling, Processing, and Storage

Whole blood was collected in EDTA tubes (BD, Franklin Lakes NJ) and centrifuged at 1900 *g* for 10 min at 4 °C within 4 h of collection. Plasma was collected and centrifuged at 2600 *g* for 15 min at 4 °C and filtered with 0.2 μm syringe filters to remove remaining platelets, apoptotic bodies, and large EVs for exosome isolation. Plasma aliquots were stored at −80 °C and centrifuged at 3000 *g* for 5 min at 4 °C to remove cryoprecipitates before use.

### Isolation of Exosomes From Plasma

Exosomes were isolated using the miRCURY Exosome Kit (Qiagen, Hilden, Germany) according to the manufacturer’s protocol. Plasma was incubated with thrombin (final concentration = 5 U/mL) for 5 min at room temperature. Plasma was centrifuged at 10,000 *g* for 5 min. Then, 1 mL of pre-treated plasma was mixed with 200 μL of precipitation buffer and incubated for 1 h at 4 °C, then centrifuged at 500 *g* for 5 min at 20 °C. The resulting pellet was resuspended in 1 mL of PBS and ultracentrifuged at 110,000 *g* for 70 min at 4 °C using the SW41Ti rotor (Beckman Coulter, Brea CA) with brakes. The supernatant was discarded, and the resulting exosome pellet was resuspended in PBS or appropriate buffers for characterisation.

### Nanoparticle Tracking Analysis (NTA)

The size distribution and concentration of exosomes were measured by nanoparticle tracking analysis (NTA) using the NanoSight NS3000 (Malvern Technologies, Malvern, UK) equipped with a 488 nm laser and CMOS camera. Samples were diluted in ultrapure water at a dilution factor of 1:100 to 1:1000 to achieve a concentration of approximately 10^7^ to 10^9^ particles/mL. Measurements were analysed from 5 × 60 s scans according to ISO guidelines (ISO/DIS19430).

### Transmission Electron Microscopy (TEM)

Freshly isolated exosome pellets were fixed in 2.5% glutaraldehyde, dropped onto a copper mesh grid, and dried at room temperature for 5 min. The samples were negatively stained with 1% ammonium molybdate for 10 s, and all excess solution was removed using filter paper. Exosomes were then imaged using the Tecnai G2 spirit transmission electron microscope (FEI, Hillsboro OR) at 100 kV.

### Total Protein and Western Blot Analysis

The samples were lysed with equal parts of RIPA buffer (Abcam, UK) and the total protein concentration was determined using the BCA protein assay kit (Pierce, Rockford, IL). An equal volume of sample was loaded and separated on a 10% SDS-PAGE gel, then transferred to a PVDF membrane. The transferred proteins were blocked with 5% milk in 0.1% TBST (Tris-buffer saline with 0.1% Tween-20) for 1 h at room temperature and incubated against primary antibodies overnight at 4 °C. The primary antibodies used were CD9 (1:1000 in 0.1% TBST plus non-fat milk, Cell Signaling Technology, Danvers MA) and TSG101 (1:1000 in 0.1% TBST plus BSA, ThermoFisher, Waltham MA). The membrane was incubated with horseradish peroxidase-conjugated anti-mouse or anti-rabbit secondary antibody (Cell Signaling Technology, Danvers MA) in 1% milk for 1 h. The proteins were detected using an enhanced chemiluminescent substrate for Western Blot [Clarity ECL kit (Bio-Rad, Hercules CA)] and captured using the ChemiDoc XRS + System (Bio-Rad, Hercules CA).

### Total Exosomal RNA Extraction

Total exosomal RNA extraction was carried out using the miRNeasy Micro Kit (Qiagen, Germany) according to the manufacturer’s protocol. Briefly, 750 μL of Qiazol was added to plasma-derived exosomes and passed through a syringe and needle five times. The homogenate was incubated for 5 min at room temperature. Chloroform was added to the homogenate to achieve phase separation after centrifugation at 12,000 *g* for 5 min at 4 °C. 1.5 volume of 100% ethanol was added to the separated aqueous phase, then passed through a spin column. The spin column was washed once with buffer RWT, buffer RPE, then 80% ethanol, before elution with 12 μL RNAse-free water. Nanodrop One (Thermo Scientific Waltham MA) and Qubit high sensitivity assay kit (Thermo Scientific Waltham MA) were used to assess total exosomal RNA concentration and purity.

### Small RNA Sequencing for miRNA Discovery

The QIAseq miRNA UDI Library Kit (Qiagen, Hilden, Germany) was used to prepare the samples used for sequencing according to manufacturer’s protocol with 5 μL of total exosomal RNA extracted from 1 mL of pre-treated plasma. Briefly, a pre-adenylated DNA adapter (1:10 dilution) was ligated to the 3′ ends of total RNA, and a 5′ RNA adapter (1:5 dilution) was ligated to 5′ end of total RNA. The ligated products were reverse-transcribed with an integrated UDI to synthesize cDNA, followed by cDNA cleanup using a streamlined magnetic bead-based method. The library was then amplified with a universal forward primer (1:10 dilution) and indexing reverse primer for 22 cycles, followed by a final library cleanup using magnetic beads. The quality and concentration of each library was assessed on LabChip GX Touch24 (Perkin Elmer, Waltham MA) and Qubit 3.0 fluorometer (Thermo Scientific Waltham MA), respectively. Samples were sequenced on the Illumina Novaseq 6000 system (Illumina, San Diego CA). Libraries were normalised to 0.5 nM and pooled before loading into the NovaSeq sequencing platform in a 1 × 75 bp single-end format. The single-end reads were aligned to the human reference genome (version hg38/GRch38) using the GeneGlobe Platform (Qiagen, Hilden, Germany).

### Bioinformatics and Differential Expression Analysis

NGS data were analysed using CLC Genomics Software version 22 (Qiagen, Hilden, Germany) using a customized pipeline. Adapters were trimmed by identifying the 3′ common sequence (AAC​TGT​AGG​CAC​CAT​CAA​T) with up to two mismatches and read length from 15 to 55 bases. Reads that were too short or did not contain the 3′ common sequence were discarded, while reads that were too long were trimmed. The filtered reads were subjected to a first round of ‘stringent’ mapping (mismatch = 0), then to a second round of ‘lenient’ mapping (mismatch = 3, and inclusion of length-based isomiRs where additions or deletions up to 2 nt were allowed) with a seed length of 18–25. Two groups of differentially expression (DE) profiles were generated, namely, (a) “stringent DE profile” with miRNA (mismatch = 0), and (b) “lenient DE profile” with miRNA (mismatch = 0-3) + isomiRs. MiRNAs with *P < 0.05* and |Log_2_ Fold Change| > 1.5 were considered differentially expressed between hypertensive and control samples. Only differentially expressed miRNAs with a maximum group mean = 20 were selected for further validation with RT-qPCR.

### Reverse Transcription of Total Exosomal RNA

Total RNA was reverse transcribed with the miRCURY LNA RT kit (Qiagen, Hilden, Germany) according to the manufacturer’s protocol. Briefly, 6 μL of template RNA was added to a reverse transcription master mix consisting of 1X miRCURY RT Reaction Buffer, 1X miRCURY RT Enzyme Mix, and RNase-free water. The reactions were incubated at 42 °C for 60 min, then at 95 °C for 5 min to inactivate the reverse transcriptase enzyme.

### Quantitative PCR (qPCR)

The qPCR reaction was setup with the pre-designed miRCURY LNA primer assays (Qiagen, Hilden, Germany) and the KAPA SYBR fast Universal kit (Roche, Wilmington MA). All PCR cycling was performed on the CFX Connect Real-Time PCR System (Bio-Rad, Berkeley, CA) using the following protocol: (i) initial PCR activation step at 95 °C, and (ii) two-step cycling of denaturation at 95 °C and annealing/extension at 56 °C between for 40 cycles. Relative quantification was calculated using the Common Base Method with primer sets U6 snRNA, hsa-miR-30d-5p, and hsa-let-7i-5p for normalisation in this study.

### Diagnostic Value of Hypertension Risk Factors and Differentially Expressed miRNA

A receiver’s operating characteristic (ROC) analysis was performed to evaluate the diagnostic value using the calculated area under the curve (AUC). The optimal cut-off value was determined by calculating the maximum Youden’s Index for statistically good ROC models (*P* < 0.05). Otherwise, the median of the hypertensive cohort expression data was used to classify samples into those with low or high miRNA expression.

### Target Prediction of Differentially Expressed miRNA

Target genes of significantly differentially expressed miRNA were predicted using three web-based programs (miRabel, miRtarbase and miRDB). A gene was considered as a potential miRNA target if it met the following criteria: (i) miRabel score <0.05, (ii) experimentally strong evidence in miRtarbase and (iii) miRDB score ≥84 according to previous studies [31, 32].

### SOMAscan Analysis

The SOMAscan assay (Somalogic, Boulder CO) was used to quantify the expression of 7000 proteins in plasma samples of three hypertensive and their matched controls to identify systemically dysregulated pathways in hypertension to support the predicted enriched pathways of differentially expressed miRNA targets. The gene set enrichment analysis (GSEA) was performed on the protein expression data with GSEA v4.3.2. The protein expression was compared against the Hallmark gene sets. Leading edge analysis was conducted on significantly dysregulated hallmarks (FDR <0.05) to identify leading edge genes.

### Gene Ontology Analysis and KEGG Pathway Enrichment

Gene ontology (GO) enrichment was performed for the analysis of biological process, cellular component, and molecular function of target genes based on the GO database (DOI: 10.5281/zenodo.7942786 Released 2023-05-10) using the PANTHER overrepresentation test (Released 13 Oct 2022) with Fisher’s Exact test. Kyoto Encyclopedia of Genes and Genomes (KEGG) pathway enrichment was conducted using the DAVID bioinformatics tool (https://david.ncifcrf.gov). The top ten enriched gene ontologies or pathways with FDR <0.05 were selected. If no enriched pathways met the criteria of FDR <0.05, *P* < 0.05 was used as criteria instead. KEGG pathways associated with cancer and infection were excluded. Additionally, functional clustering with the DAVID bioinformatics tool was conducted on the enriched GO and KEGG pathways of leading-edge genes from GSEA analysis of circulating proteins.

### Visualization of miRNA-Target Gene and Protein-Protein Interaction Network

All networks were visualized with Cytoscape v3.10.0 software. The predicted KEGG pathways from DAVID analysis (FDR <0.05 or *P* < 0.01) were summarized to produce enriched gene sets (enrichment map) with the EnrichmentMap plugin on Cytoscape. The genes involved in the enrichment map were extracted, and the number of shared pathways were computed for each gene to determine overrepresented genes. MiRNA-target gene interactions from the enrichment map were visualized.

### Statistics Analysis

IBM SPSS v27.0 (SPSS inc., Chicago IL) and GraphPad Prism 8.0 (GraphPad Software, San Diego CA) were used to analyse and display data. Descriptive analysis was conducted on demographic variables between hypertensives and controls. Unpaired student’s t-test, Chi-square test or Mann-Whitney *U* test was performed where appropriate to determine the significance between groups. *P* < 0.05 was considered statistically significant.

## Results

### Measures of Obesity Significantly Increased in Hypertensives

The demographics of participants involved in the study are summarised in Table 1. Participants were newly diagnosed hypertensives between 30 and 55 years old (mean = 44 ± 7.49). Healthy controls were generally overweight (mean BMI = 25.7 ± 4.8), while hypertensives were predominantly obese (mean BMI = 30.0 ± 3.8) with abdominal obesity and a significantly higher total cholesterol (TC)/high density lipoprotein (HDL) ratio (*P* < 0.05).

**TABLE 1:** Demographics and clinical characteristics of controls and hypertensives (n = 35). SBP, DBP, BMI, waist circumference and TC/HDL ratio were significantly higher in hypertensives than controls. Data expressed as mean ± SD or n (%).

Variables	Control (n = 17)	Hypertensive (n = 18)	*P*-value
Age (years)	40.5 ± 7.5	44.0 ± 7.5	0.179
Sex			
Male	9 (52.9)	10 (55.6)	0.615^a^
Female	8 (47.1)	8 (44.4)	
Race			
Malay	11 (64.7)	11 (61.1)	0.826^a^
Chinese	6 (35.3)	7 (38.9)	
Family history of hypertension			
No	4 (23.5)	1 (5.6)	0.129^a^
Yes	13 (76.5)	17 (94.4)	
SBP (mmHg)	120.4 ± 9.9	148.1 ± 7.4	<0.001^b^ ^,^ ^c^
DBP (mmHg)	81.5 ± 8.1	91.2 ± 9.3	<0.001^b^ ^,^ ^c^
BMI (kg/cm^2^)	25.7 ± 4.8	30.0 ± 3.8	0.001^b^ ^,^ ^c^
WC			
Normal	9 (52.9)	3(16.7)	0.024^a^ ^,^ ^c^
Abdominal obesity	8 (47.1)	15 (83.3)	
TC (mg/dL)	5.7 ± 0.9	5.9 ± 1.0	0.308^b^
HDL (mg/dL)	1.3 ± 0.3	1.3 ± 0.3	0.426^b^
LDL (mg/dL)	3.4 ± 0.9	3.9 ± 0.7	0.125^b^
TG (mg/dL)	1.7 ± 1.4	1.7 ± 1.2	0.729^b^
TC/HDL ratio	4.2 ± 1.6	4.8 ± 1.1	0.031^b^ ^,^ ^c^
Plasma glucose	4.7 ± 0.8	5.2 ± 0.8	0.080^b^

^a^ Chi-square test.

^b^ Mann-Whitney *U* test.

^c^ Indicates P < 0.05 using Chi-square test or Mann-Whitney *U* test as appropriate.

SBP, systolic blood pressure; DBP, diastolic blood pressure; BMI, body mass index; WC, waist circumference; TC, total cholesterol; HDL, high density lipoprotein; LDL, low density lipoprotein; TG, triglyceride.

### Hypertensives Have Significantly Decreased Exosomal CD9 Expression

**FIGURE 1:**
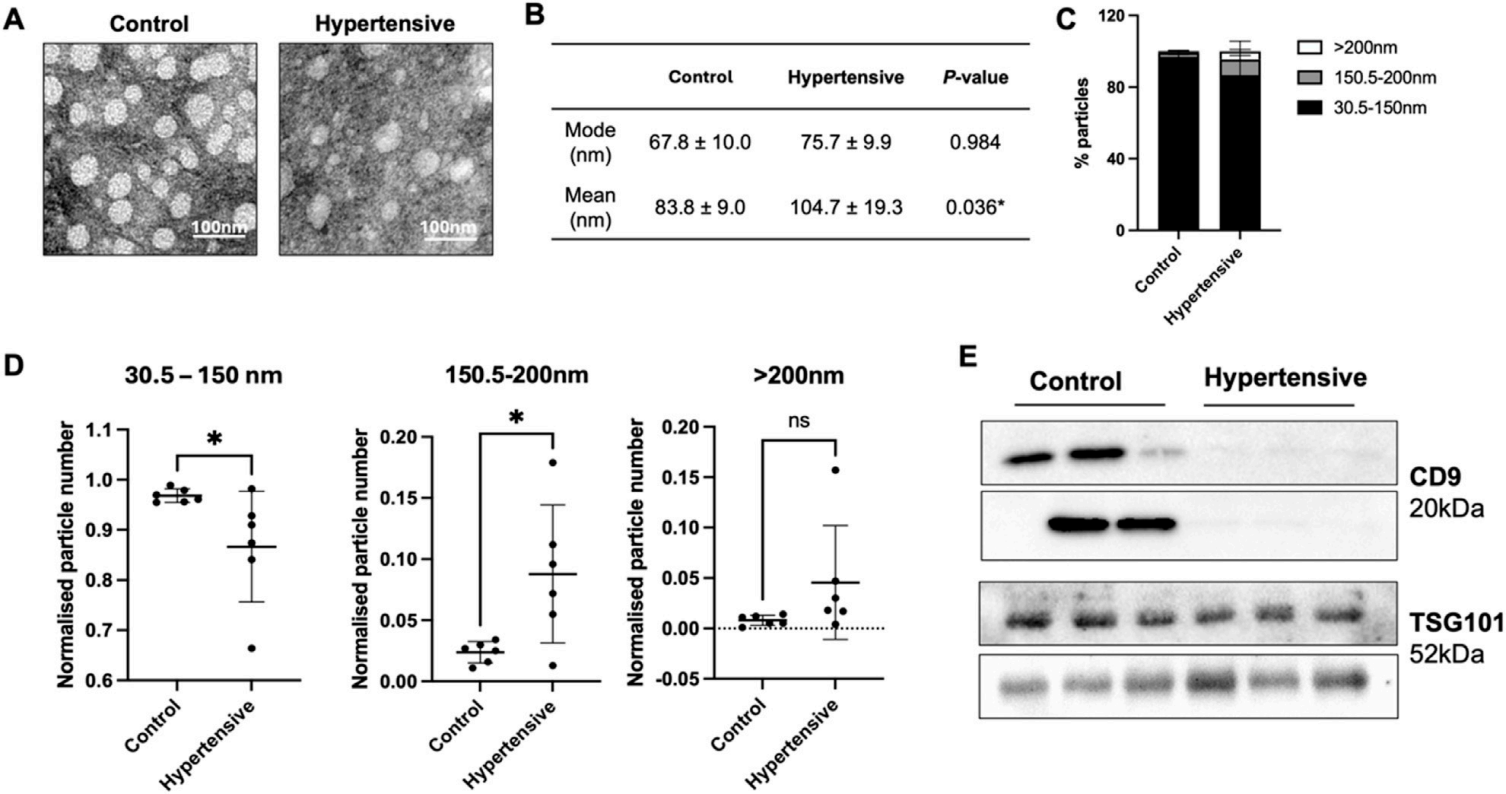
Characterization of plasma exosomes from hypertensives and controls (n = 12). Characterization of plasma exosomes was conducted on the six hypertensives and their respective controls that underwent next-generation sequencing. Vesicle-shaped morphologies were observed using TEM **(A)**, while size and particle distribution was assessed with nanoparticle tracking analysis **(B–D)**. All measurements using nanoparticle tracking analysis were analysed from 5 × 60 s scans according to ISO guidelines. Immunoblotting with exosome markers, CD9 and TSG101, were used to confirm the presence of exosomes from each of the twelve samples **(E)**. The isolated vesicles were within the range of typical exosomes **(B)**, but hypertensives preferentially released vesicles larger than 150 nm **(C,D)**, and exhibited low to no expression of CD9^+^ exosomes **(E)**. * Indicates *P* < 0.05 statistical difference using student’s t-test.

Plasma exosomes were isolated using a combined precipitation and ultracentrifugation method. Distinct vesicle-shaped structures were consistently observed, exhibiting a round and uniform appearance using TEM (Figure 1A) with characteristic size of exosomes (Figures 1B,C), indicating successful isolation of exosomes There was no significant difference in overall mode size and particle number between controls and hypertensives (Figures 1B,C; Supplementary Table S1).

We then analysed the number of particles released according to three size classes, small EVs (30–150 nm), medium EVs (150.5–200 nm) and large EVs (>200 nm). Hypertensives exhibited a higher proportion of medium and large EVs (Figure 1C), significantly increasing its mean size (Figure 1B). When normalised to the total number of particles, there was a significant reduction in the number of small EVs and an increase in the number of medium EVs released by hypertensives (Figure 1D). Moreover, hypertensives released a significantly lower amount of CD9^+^ exosomes with no difference in TSG101^+^ exosomes compared to controls (Figure 1E).

### Primary and Secondary Analyses of Small RNA Sequencing Data

The total number of reads obtained after sequencing was 99,189,544 reads with an average of 8,265,795 ± 1,624,737 reads per sample with an average Q score of 35.8%, indicating a successful sequencing run (Supplementary Table S2). All the retained reads were of good quality (Q score = 30 - 40) with negligible ambiguous base content. After adapter trimming, on average, 51.5% ± 19.8% of retained reads were mapped to miRbase. Due to the short length of miRNAs, an average of 19.5% ± 8.4% of the retained reads were ambiguously annotated and discarded, resulting in the loss of data from nearly half of the mapped reads (Supplementary Table S3). A second round of mapping using a lenient criterion was employed to overcome the low mapping statistics. The lenient criteria included mapping to mature miRNAs up to three mismatches and the addition of up to two nucleotides at the 5′ and 3′ ends for the detection of isomiRs. Only a minute number of reads (<3.50%) mapped to mature miRNAs with more than one mismatch or mapped to isomiRs in both controls and hypertensives (Supplementary Figure S1). Due to challenges in read mapping, it was pertinent that validation of the sequencing results was conducted with qPCR.

### Inclusion of isomiRs Influence Differential Expression Profile

To rule out profiles that might be masked by noise such as sequencing error, two differential expression (DE) profiles from stringent mapping and lenient mapping were conducted. The stringent DE profile referred to differential expression of miRNA without mismatches (mismatch = 0), and the lenient DE profile referred to miRNA up to three mismatches (mismatch = 0–3) with the inclusion of length-based isomiRs.

**FIGURE 2:**
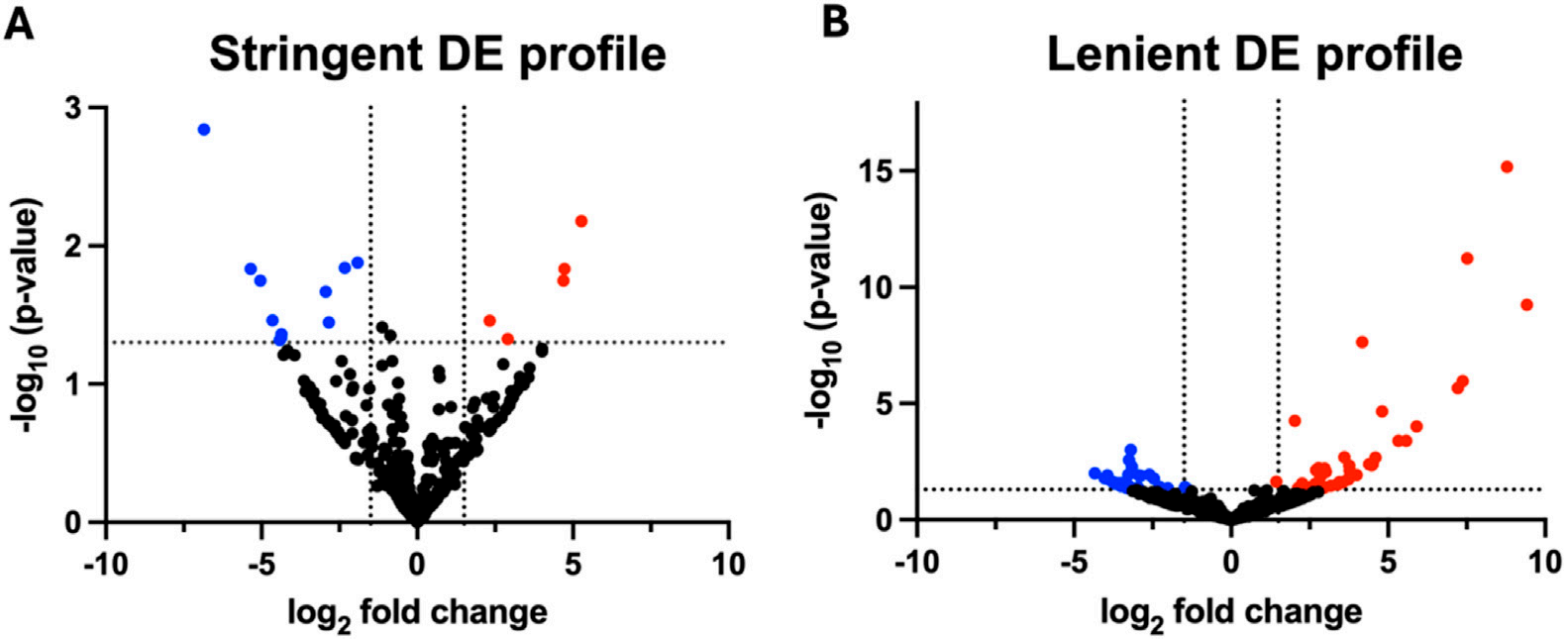
Volcano plots of differential expression profiles (n = 12). Volcano plots of differential miRNA profiles were generated from six hypertensive patients and their respective controls that underwent next-generation sequencing. Differentially expressed miRNA of the **(A)** stringent DE profile yielded 11 downregulated and 5 upregulated miRNA, whereas **(B)** the lenient DE profile yielded 31 downregulated and 39 upregulated miRNA. Stringent DE profile was obtained from the DE analysis of reads mapped to miRNAs with no mismatches. Lenient DE profile was obtained from the DE analysis of reads mapped to miRNAs with up to three mismatches and length-based isomiRs. MiRNAs were considered differentially expressed when *P* < 0.05 and log_2_FC |1.5|. *P*-values were calculated using standard statistical analyses methods in the CLC Genomics differential expression pipeline. DE, differential expression.

A total of 16 and 70 miRNAs were differentially expressed in the stringent DE profile (Figure 2A) and lenient DE profile (Figure 2B), respectively, when *P* < 0.05 and Log_2_FC |1.5|. Nearly 70% of the differentially expressed miRNAs (11 of 16) of the stringent DE profile were downregulated. In contrast, the miRNA lenient DE profile exhibited a more balanced distribution, where 44.3% (31 of 70) of differentially expressed miRNAs were downregulated and 55.7% (39 of 70) were upregulated.

**FIGURE 3:**
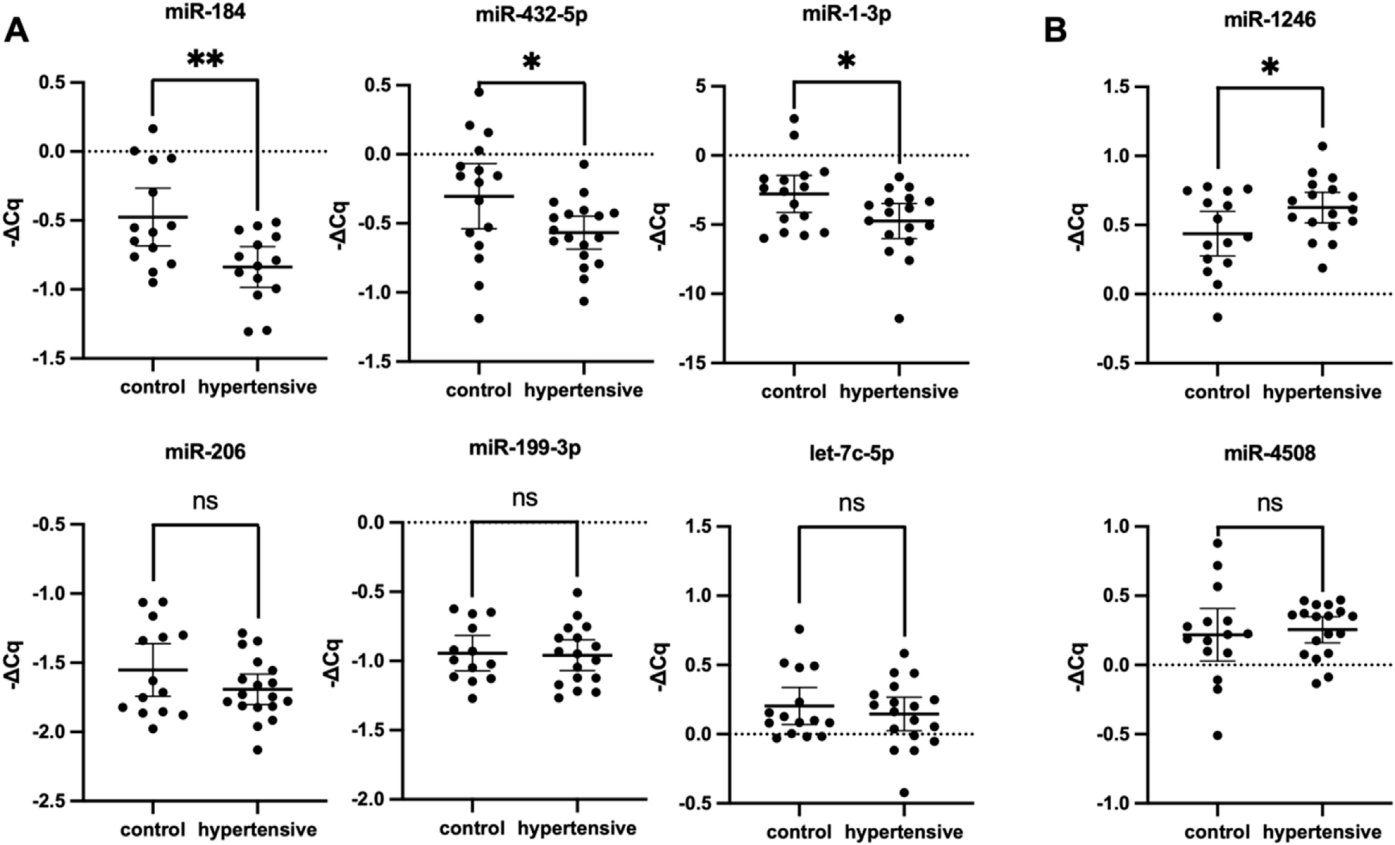
Validation of differentially expressed miRNA RT-qPCR (n = 35). Hsa-miR-184, hsa-miR-432-5p and hsa-miR-1-3p were significantly downregulated **(A)**, whereas miR-1246 was significantly upregulated **(B)**. Expression levels of miRNA were normalised to U6, hsa-miR-30d-5p, and hsa-let-7i-5p. Housekeeping genes were selected from literature and based on stable expression in NGS data. Data expressed as mean ±95% CI, with two technical replicates per biological replicate. The hypertensive group consisted of 18 biological replicates and the control group included 17. * indicates *P < 0.05* statistical difference using student’s t-test or Mann-Whitney *U* test as appropriate. ns, not significant; CI, confidence interval.

The variability in DE profile when isomiRs and mismatched sequences were included in the bulk sequencing signal suggested high isomiR diversity in hypertensives. Moreover, the opposing trends in small RNA-seq versus qPCR DE profiles support the increased isomiR diversity in hypertensives. For example, the isomiR diversity in hypertensives were lost with qPCR validation due to the inability of current qPCR methods to detect miRNA variations, resulting in downregulation of hsa-miR-184 and hsa-miR-1-3p in qPCR (Figure 3), but upregulation in the small RNA-Seq DE analysis (Table 2). Due to limitations in the current bioinformatics pipeline and limitations in isomiR validation technologies, a more in-depth analysis of isomiRs could not be conducted. Therefore, this study only aimed to validate DE profiles of canonical miRNA.

**TABLE 2:** Significantly differentially expressed miRNA from small RNA sequencing data (n = 12). MiRNAs were considered significantly differentially expressed when *P* < 0.05, log_2_FC |1.5|, and max group mean >20.

miRNA	Max group mean	log_2_FC	*P*-value	FDR-value
Downregulated miRNA				
hsa-miR-432-5p	83.8	−1.91	0.010	0.760
hsa-miR-199a-3p	22.5	−2.33	0.010	0.760
Upregulated miRNA				
hsa-miR-3960	43.8	9.41	<0.0001	<0.0001
hsa-miR-4508	98.7	8.78	<0.0001	<0.0001
hsa-miR-1246	51.0	7.51	<0.0001	<0.0001
hsa-miR-1-3p	123.0	4.17	<0.0001	<0.0001
hsa-miR-184	280.1	3.61	0.002	0.050
hsa-miR-206	275.3	2.78	0.006	0.100
hsa-let-7c-5p	1,150.2	2.03	0.000	0.003

Filtering differentially expressed miRNAs from the stringent and lenient DE profile by the maximum group mean resulted in a final list of nine differentially expressed miRNAs. P-value was calculated using standard statistical analyses methods in the CLC Genomics differential expression pipeline Negative (−) log_2_FC indicated downregulation. DE, differential expression; log_2_FC, fold change; FDR, false discovery rate.

### Differential Expression Profile Identified From Exosomal miRNA in Hypertensives

Considering that perfectly mapped miRNAs contributed to almost all mapped miRNA reads and a more desirable FDR-value was obtained when mapping conditions were more lenient, DE profiles from both groups were considered in the selection of miRNAs for validation. To address the limitations posed by low mapping and to mitigate potential results influenced by sequencing errors, miRNAs with a maximum group mean greater than 20 (max group mean >20) were selected for further validation using RT-qPCR. Filtering the DE profile based on this metric ensures retention of genes with meaningful expression differences between sample groups while excluding those with weak signals in both groups and revealed nine differentially expressed exosomal miRNAs (Table 2). Among these, hsa-miR-432-5p and hsa-miR-199a-3p were downregulated, while the remaining six differentially expressed miRNAs were upregulated. In contrast, RT-qPCR relative expression levels demonstrated that among the nine miRNA candidates, six miRNAs were downregulated, and two miRNAs were upregulated (Figure 3) due to the inability of primers to detect isomiRs. Statistically significant differences in miRNA expression were observed for hsa-miR-1-3p, hsa-miR-184, hsa-miR-432-5p, and hsa-miR-1246 with a fold change of −1.495 (*P* = 0.031), −2.302 (*P* = 0.005), −1.836 (*P* = 0.036), and 1.748 (*P* = 0.044), respectively. The expression of hsa-miR-3960 was not evaluated because of the high GC content (>95%) led to the production of non-specific qPCR products.

### Diagnostic Value of Differentially Expressed Exosomal miRNA

**FIGURE 4:**
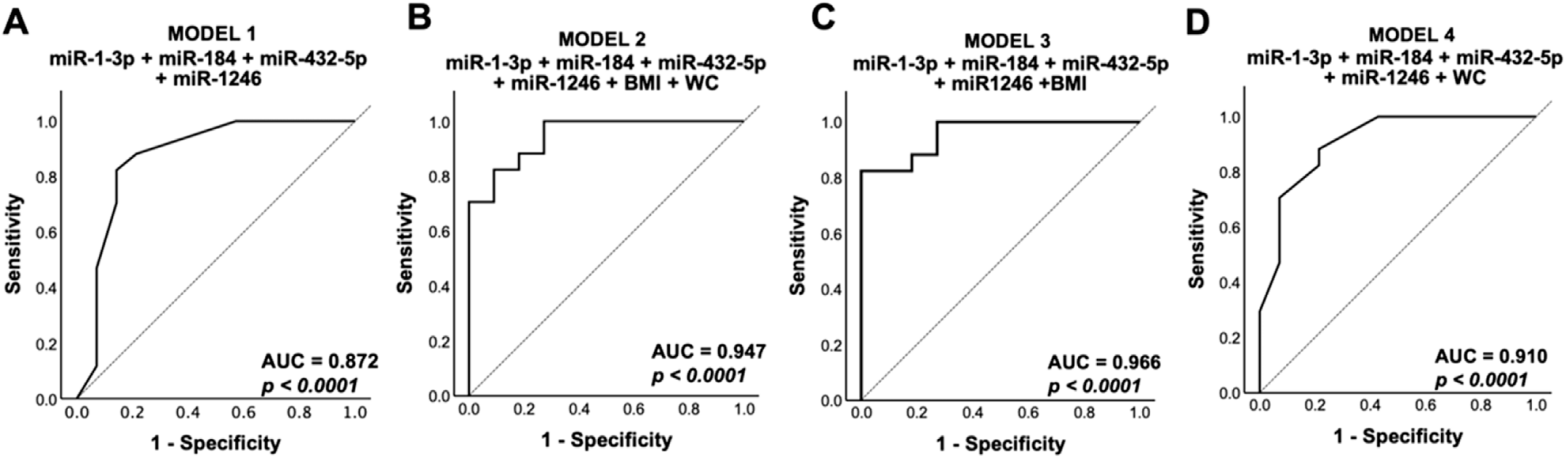
Receiver operating characteristic analysis. Predicted probabilities from multivariate analysis of **(A)** model 1: sig. DE miRNA, **(B)** model 2: sig. DE miRNA + BMI + WC, **(C)** model 3: sig. DE miRNA + BMI, and **(D)** model 4: sig. DE miRNA + WC from the qPCR validation group (n = 18 for hypertensives; n = 17 for control groups, where n refers to biological replicates) were used to generate the receiver operating curve. Sig. DE miRNA refers to significantly differentially expressed miRNA from qPCR validation (hsa-miR-1-3p, hsa-miR-184, hsa-miR-432-5p, and hsa-miR-1246). Model 3 showed the highest diagnostic value, with the highest AUC. DE, differentially expressed; BMI, body mass index; WC, waist circumference; AUC, area under curve.

ROC analysis was conducted on four models comprising various combinations of the significant differentially expressed miRNAs and distinct risk factors such as BMI and abdominal obesity. The results showed that all models demonstrated excellent discriminatory power, with an AUC value exceeding 0.8 (*P* < 0.0001) (Figure 4), surpassing the diagnostic power of individual miRNAs (Supplementary Table S4). However, the results further indicated that the inclusion of BMI with the significant DE miRNAs (model 3) (Figure 4) exhibited the best diagnostic value by improving specificity, thereby enhancing the ability to distinguish between hypertensives and healthy controls.

### Downregulated Exosomal miRNAs Are Associated With Inflammation and Vascular Remodelling, Hormone Synthesis and Secretion, and Regulation of Metabolic Processes

**FIGURE 5:**
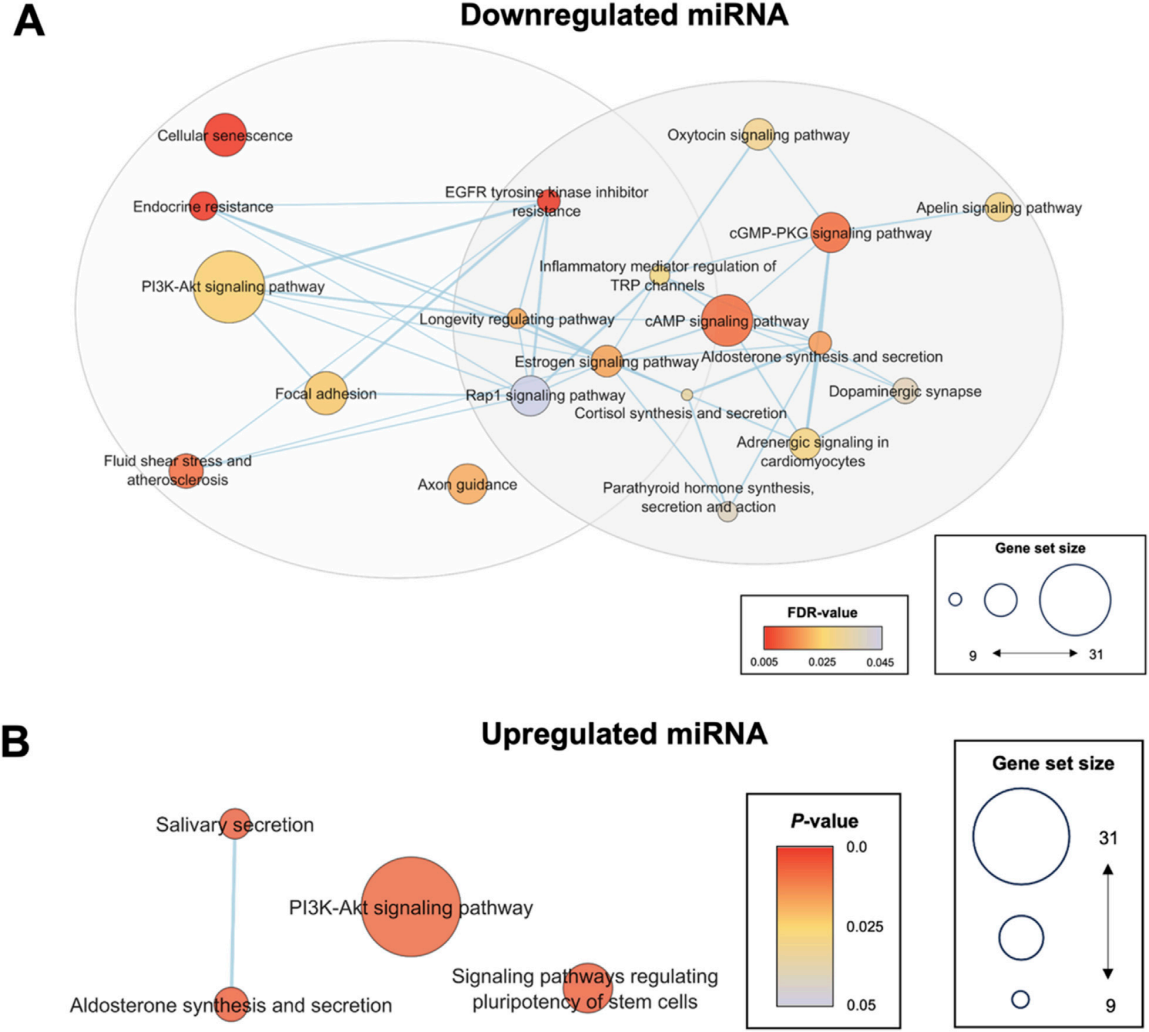
Enrichment map of enriched pathways from significantly differentially expressed miRNA targets. KEGG pathway enrichment analysis was conducted using the standard pipeline in the DAVID bioinformatics tool. All KEGG pathways with FDR <0.05 or *P* < 0.05 for the validated downregulated or upregulated miRNA targets, respectively, were selected to generate the pathway enrichment map for visualization of relationships between the enriched pathways and reduce redundancy between terms. Pathways associated with downregulated targets were clustered into two groups associated with (i) inflammation and vascular remodelling, and (ii) hormone synthesis and secretion **(A)**. No significantly enriched pathways were identified for upregulated miRNA when a cut-off of FDR <0.05 was used **(B)**. Hence, *P* < 0.05 was utilized as the cut-off point for upregulated miRNA. Sig. DE, significantly differentially expressed; KEGG, Kyoto Encyclopedia Genes and Genomes.

To further corroborate the selection of hsa-miR-1-3p, hsa-miR-184, hsa-miR-432-5p, and hsa-miR-1246 as biomarker candidates of hypertension, its associated gene ontologies and pathways were compared against systemically dysregulated pathways to determine its functional importance. KEGG pathway enrichment analysis of hsa-miR-1-3p, hsa-miR-184, hsa-miR-432-5p target mRNAs clustered into two groups based on gene set similarity (Figure 5A). Cluster 1 was associated with inflammation and vascular remodelling, and included pathways such as cellular senescence and PI3K/AKT signalling. Cluster 2 was associated with hormone synthesis and secretion, and included key regulators known to modulate blood pressure such as cortisol and aldosterone synthesis and secretion, and oxytocin, dopaminergic and adrenergic signalling. Targets of downregulated miRNA were enriched in biological processes such as monocyte homeostasis, cardiac vascular smooth muscle cell differentiation, regulation of vascular smooth muscle cell apoptotic processes, and regulation of cell growth in cardiac muscle development (Supplementary Figure S2A). This is consistent with the understanding that the development of hypertension involves increased inflammation, differentiation and apoptosis of vascular or cardiac cells. The components of the neural system, especially the semaphorin-plexin signalling pathway in axon guidance, were significant in all gene ontology subcategories, suggesting its role in the development of hypertension (Supplementary Figure S2A). Furthermore, overrepresented genes of the miRNA-mRNA network were isoforms such as *PRKCB* and *AKT2* that are typically involved in regulating metabolism through the PLC/PKC or PI3K/AKT pathway (Supplementary Figure S3A). These results emphasised a potential key role of exosomal miRNA in driving various metabolic disruptions and vascular dysfunctions that contribute to increased blood pressure.

### Upregulated Exosomal miRNAs Associated With VEGF Signalling and Regulation of Heart Contraction Through Calcium Signalling

Targets of the upregulated hsa-miR-1246 were enriched in VEGFR2 binding, PI3K/AKT signalling, and positive regulation of phosphatase activity (Figure 5B). These targets also involved kidney development, negative regulation of apoptotic pathways, BMP signalling, angiogenesis, and heart contraction (Supplementary Figure S2B). The overrepresented genes in this miRNA-mRNA network were associated with the calcium signalling pathway and the regulation of calcium reabsorption (Supplementary Figure S3B). The findings suggest that hsa-miR-1246 plays a role in the interaction between the nervous system, suppression of kidney development, and regulation of heart contraction, highlighting its potential impact on vascular and cardiac functions.

### GSEA Analysis of Circulating Proteins Revealed Altered Metabolism in Hypertensives

GSEA revealed hallmarks of impaired metabolism in hypertensives (Supplementary Table S5). Specifically, there was downregulation of pathways related to PI3K/AKT/MTOR signalling, Myc targets, fatty acid metabolism, oxidative phosphorylation, and the ROS pathway. The functional clustering of leading-edge genes revealed ten distinct clusters associated with pathways related to peroxisomes, fatty acid β-oxidation, translation initiation, diabetic cardiomyopathy or response to ROS, protein folding and binding, glycolytic processes, apoptosis related protein insertion into mitochondrial membrane, tricarboxylic acid (TCA) cycle, cell redox homeostasis, and propanoate metabolism (Table 3). Gene ontologies highlighted dysregulation of mitochondrial metabolic processes and collectively suggest alterations in TCA metabolites, impaired mitochondrial function, and overall altered metabolism in hypertensives.

**TABLE 3:** Functional clustering of gene ontology and KEGG terms (n = 6). Leading edge genes of downregulated hallmarks in circulating proteins of three newly diagnosed hypertensives and their respective controls were subjected to pathway enrichment analysis. Top 10 clusters were reported. All statistics were generated using the standard gene ontology and KEGG analysis pipeline using the DAVID bioinformatics tool.

Cluster	Category	Term	Fold	*P*-value	FDR
1	Enrichment score: 5.7				
	GO CC	Peroxisomal matrix	17.6	3.8 × 10^8^	1.5 × 10^6^
	GO CC	Peroxisome	9.1	1.6 × 10^6^	5.0 × 10^5^
	KEGG	Peroxisome	5.7	1.6 × 10^4^	9.4 × 10^4^
2	Enrichment score: 5.0				
	GO BP	Fatty acid beta-oxidation	23.1	2.9 × 10^12^	4.9 × 10^9^
	KEGG	Valine, leucine and isoleucine degradation	12.1	1.5 × 10^8^	8.1 × 10^7^
	KEGG	Fatty acid degradation	9.8	1.4 × 10^5^	1.7 × 10^4^
	KEGG	Fatty acid metabolism	7.4	8.9 × 10^5^	6.4 × 10^4^
	KEGG	Butanoate metabolism	11.7	1.3 × 10^4^	8.6 × 10^4^
	KEGG	Tryptophan metabolism	6.3	7.8 × 10^3^	1.6 × 10^2^
	KEGG	Lysine degradation	3.3	1.2 × 10^1^	1.3 × 10^1^
3	Enrichment score: 5.0				
	GO BP	Translational initiation	15.8	9.1 × 10^8^	7.8 × 10^5^
	GO MF	Translation initiation factor activity	13.8	2.6 × 10^7^	1.9 × 10^5^
	GO CC	Eukaryotic translation initiation factor 4F complex	33.9	1.9 × 10^4^	3.7 × 10^3^
	GO MF	Translation factor activity, RNA binding	16.2	1.8 × 10^3^	3.8 × 10^2^
4	Enrichment score: 3.9				
	KEGG	Diabetic cardiomyopathy	4.7	2.4 × 10^7^	7.7 × 10^6^
	KEGG	Pathways of neurodegeneration - multiple diseases	2.9	2.7 × 10^6^	6.2 × 10^5^
	KEGG	Chemical carcinogenesis - reactive oxygen species	4.0	4.3 × 10^6^	8.6 × 10^5^
	KEGG	Alzheimer disease	2.9	3.4 × 10^5^	3.2 × 10^4^
	KEGG	Prion disease	3.3	5.2 × 10^5^	4.1 × 10^4^
	KEGG	Parkinson disease	3.2	1.4 × 10^4^	8.8 × 10^4^
	KEGG	Sphingolipid signalling pathway	4.4	4.5 × 10^4^	1.8 × 10^3^
	KEGG	Non-alcoholic fatty liver disease	3.7	6.8 × 10^4^	2.4 × 10^3^
	KEGG	Amyotrophic lateral sclerosis	2.5	1.4 × 10^3^	3.9 × 10^3^
	KEGG	Huntington disease	2.4	5.1 × 10^3^	1.1 × 10^2^
	KEGG	Dopaminergic synapse	2.8	3.8 × 10^2^	5.5 × 10^2^
5	Enrichment score: 3.9				
	GO MF	Unfolded protein binding	9.2	1.8 × 10^8^	1.9 × 10^6^
	GO BP	Protein folding	6.2	3.7 × 10^6^	1.0 × 10^3^
	GO MF	Protein binding involved in protein folding	11.2	1.9 × 10^4^	6.8 × 10^3^
	GO BP	Protein stabilization	3.8	2.8 × 10^3^	1.3 × 10^1^
	GO BP	Response to unfolded protein	8.4	2.8 × 10^3^	1.3 × 10^1^
	GO MF	ATPase activity	2.1	4.8 × 10^2^	3.5 × 10^1^
6	Enrichment score: 3.3				
	GO BP	Glycolytic process	12.3	1.2 × 10^4^	1.6 × 10^2^
	KEGG	Biosynthesis of amino acids	5.6	5.0 × 10^4^	1.9 × 10^3^
	KEGG	Glycolysis/Gluconeogenesis	5.5	1.6 × 10^3^	4.3 × 10^3^
7	Enrichment score: 2.8				
	GO BP	Positive regulation of protein insertion into mitochondrial membrane involved in apoptotic signalling pathway	48.2	2.2 × 10^6^	7.6 × 10^4^
	KEGG	PI3K-Akt signalling pathway	3.1	1.3 × 10^5^	1.7 × 10^4^
	GO BP	Substantia nigra development	12.0	1.3 × 10^4^	1.6 × 10^2^
	KEGG	Hepatitis C	4.0	1.9 × 10^4^	1.1 × 10^3^
	GO MF	Protein domain specific binding	4.1	3.8 × 10^4^	1.2 × 10^2^
	KEGG	Viral carcinogenesis	3.1	1.6 × 10^3^	4.4 × 10^3^
	KEGG	Cell cycle	3.0	9.6 × 10^3^	1.9 × 10^2^
8	Enrichment score: 2.8				
	GO BP	Tricarboxylic acid cycle	13.8	4.5 × 10^4^	3.7 × 10^2^
	KEGG	Citrate cycle (TCA cycle)	8.8	2.3 × 10^3^	5.6 × 10^3^
	GO BP	Malate metabolic process	28.9	4.5 × 10^3^	1.8 × 10^1^
9	Enrichment score: 2.7				
	GO BP	Cell redox homeostasis	10.9	1.1 × 10^3^	6.9 × 10^2^
	GO MF	Thioredoxin peroxidase activity	40.0	2.3 × 10^3^	4.4 × 10^2^
	GO MF	Peroxiredoxin activity	40.0	2.3 × 10^3^	4.4 × 10^2^
10	Enrichment score: 2.4				
	KEGG	Propanoate metabolism	9.9	3.0 × 10^4^	1.4 × 10^3^
	GO BP	Branched-chain amino acid catabolic process	22.2	7.7 × 10^3^	2.2 × 10^1^
	KEGG	Beta-Alanine metabolism	6.8	2.0 × 10^2^	3.4 × 10^2^

In addition, upregulated pathways in pancreatic beta cells suggest increased insulin secretion. While the functional analysis of leading-edge genes in upregulated hallmarks did not show significant clusters, KEGG analysis indicated upregulation of insulin signalling (Supplementary Figure S4A). Gene ontologies revealed enrichment in glycolysis, glucose metabolism, and glycogen biosynthesis, suggesting a caloric surplus due to an imbalance between energy intake and expenditure, typical of obesity (Supplementary Figure S4B).

## Discussion

Our study identified distinct miRNA expression profiles in hypertensives, with notable differences between stringent and lenient mapping criteria. Our study also found that in newly diagnosed and untreated hypertensive patients, there were specific changes in exosome size distribution and decreased CD9 expression. There was an increased proportion of vesicles larger than 150 nm (Figure 1). Consistent with the findings of this study, current research on small EV profiles found no significant differences in overall size and concentration between hypertensives and controls (Figure 1B, Supplementary Table S2) [33, 34]. However, there is growing evidence indicating an increased release of endothelial- or platelet-derived EVs (100-1000 nm) in hypertensives [35, 36]. Specifically, endothelial-derived EVs (CD105^+^ EVs) in hypertensive animal models increased significantly, with sizes ranging from 120 nm to 200 nm when measured with NTA and cryo-electron microscopy [37]. The significant increase in EVs larger than 150 nm in hypertensives when normalised to total number of particles in this study (Figure 1D) likely reflects this elevated release, leading to more sedimentation of larger vesicles during ultracentrifugation. More research is needed to characterise the phenotypes and origins of EVs beyond only exosomes, including medium and larger microvesicles, and elucidate their biological importance in hypertension development.

In this study, the tetraspanin, CD9, often enriched in the exosome membrane and at the cellular plasma membrane, was chosen as a positive marker due to its role in exosome release, uptake, and its abundance in plasma compared to other commonly used tetraspanins [38–41]. Conversely, TSG101 has been implicated in exosome biogenesis through the endolysosomal pathway via ESCRT machinery [42, 43]. The significantly reduced CD9 expression with unchanged TSG101 levels in hypertensive exosomes (Figure 1E) raises interesting questions about EV surface markers and their role in hypertension. Previous studies noted a modest and statistically insignificant decrease of exosomal CD9 in treated hypertensives, potentially due to the effects of anti-hypertensive medication [33, 34]. CD9, now recognised as a new marker of senescence, is typically increased in aged arteries and atherosclerotic plaques [44]. Research has consistently shown that hypertension is closely linked to vascular aging, which its primary processes include cellular senescence [45–48]. Other than a positive marker for exosomes, CD9 is now recognised as a new marker of senescence. It has been found to induce cellular senescence and aggravate atherosclerotic plaque formation through the phosphatidylinositide 3 kinase-AKT-mTOR-p53 signal pathway, alter VSMC phenotypes and regulate inflammation [44, 49]. While CD9 is found to be typically increased in aged arteries and atherosclerotic plaques itself, pathways to increased tissue expression is not known [44]. Considering the emerging importance of CD9 in senescence, significant changes in exosomal CD9 expression in hypertensives indicate a novel insight to its potential links to vascular aging and hypertension that have yet to be uncovered. In a previous study, older adults (>70 years) showed reduced CD9 expression in plasma exosomes, suggesting a possible shift of CD9 from exosomes to tissues, and implicated vascular aging in newly diagnosed hypertensives of this study [50, 51]. Mechanisms resulting in opposing trends in CD9 expression between vascular tissues and exosomes have not yet been elucidated. Recent studies showed that CD9 is functionally redundant in the release of exosomes as its knockdown was compensated by CD63 during its biogenesis [41, 52]. It is plausible that senescence-inducing stimuli might trigger the redistribution of CD9 to tissues rather than its participation in exosome biogenesis, potentially influencing the subtype of EVs released and the pathophysiology of vascular aging and hypertension. Elucidating these mechanisms requires additional research efforts to unravel the complexities of CD9 dynamics in the context of vascular aging and hypertension. These findings further indicate that exosomal CD9 is a promising novel target for diagnostics or therapeutics.

The study identified four miRNAs (hsa-miR-1-3p, hsa-miR-184, hsa-miR-432-5p, and hsa-miR-1246) with significant differential expression in hypertensives (Figure 3). These miRNAs, particularly when combined with BMI, demonstrated excellent diagnostic potential for hypertension, suggesting their potential use as biomarkers (Figure 4). As population studies showed that obesity contributed to more than 65% of all essential hypertension cases, it was unsurprising that measures of obesity were vital in predicting the risk of hypertension [53]. In this study, the discrepancies between miRNA profiling and validation results suggested the inability of current qPCR methods to detect miRNA variations. The loss in isomiR diversity was evident in hsa-miR-184 and hsa-miR-1-3p which were downregulated in qPCR but upregulated in the small RNA-Seq DE analysis. IsomiR profiling in plasma exosomes showed that nucleotide substitutions most frequently occurred in exosomal miRNA at the 12 and 14 position of the miRNAs [54, 55]. Moreover, 3′ uridylation had been suggested as a mechanism for miRNA loading into exosomes [56]. These 3′-end modifications commonly found in exosomal miRNA raise an interesting research question on the role of hsa-miR-184 and hsa-miR-1-3p isomiRs in the development of hypertension, which is not well understood. Given distinct variation patterns in exosomal miRNA, isomiRs add another layer of complexity to the analysis of exosomal miRNAs which is worth exploring in the future to accurately elucidate the molecular functions between gene silencing components in disease regulation.

Predicted target pathways of this miRNA signature were associated with fundamental cellular and molecular mechanisms of aging, including oxidative stress, chronic low-grade inflammation and vascular remodelling, supporting alterations of the senescence marker, CD9, in the exosomes of hypertensives (Figure 5) [57, 58]. Previous studies had found the association of these miRNAs to various cardiovascular diseases. For example, the downregulation of miR-1-3p and miR-432-5p was shown to drive heart failure and myocardial ischemic injury by regulating pathways related to oxidative stress, while the upregulation of miR-1246 promotes VEGF signalling associated with angiogenesis, proliferation, and vascular remodelling [59–62]. In turn, the downregulation of miR-184 has been associated with obesity-related inflammation, insulin secretion, and resistance through aberrant PKCβ and AKT2 signalling [63–65].

Proteomics data in this study demonstrated systemic downregulation of pathways and processes associated with mitochondria, impaired fatty acid metabolism, and increased insulin signalling in hypertensives, often observed in obesity-driven cardiovascular damage (Table 3, Supplementary Figure S4) [66–69]. These findings aligned with previous studies where mitochondrial and metabolic abnormalities were observed in hypertension models, and supported indications of obesity-related hypertension [70, 71]. Prospective clinical studies have shown that measures of vascular aging were significantly increased and positively associated with obesity and elevated blood pressure, suggesting the role of vascular aging as an underlying driver of hypertension [45–47]. Therefore, overall data suggests the role of overactive miRNA targets from the hypertension signature in driving vascular damage during obesity-related hypertension development. Furthermore, these results demonstrate the biological importance of hsa-miR-184, hsa-miR-432-5p, hsa-miR-1-3p, and hsa-miR-1246 in hypertension, further strengthening their use as biomarkers.

The limitations of this study included the small sample size, challenges in small RNA sequencing of exosomal miRNA, and limitations of isomiR validation technology. Additionally, the results of this study were specific to those who were already hypertensive with some signs of metabolic syndrome. Therefore, these results cannot be generalised to hypertensives with the other non-communicable diseases. Larger longitudinal studies on pre-hypertensive patients are needed to further develop the use of the identified exosomal miRNA signature as a predictive biomarker to stratify the risk of developing hypertension. Despite these limitations, it was necessary to investigate patients with solely hypertension to demonstrate the association between exosomal miRNA and hypertension within the Malaysian population as no previous studies had been conducted in this area. These results would aid in the interpretation of future miRNA studies in larger cohorts, supporting the development of predictive hypertension biomarkers and facilitating its translation into clinical practice.

In conclusion, this study provides novel insights into the exosomal miRNA profile of hypertensive individuals and demonstrate the association between exosomal miRNA and hypertension. The findings highlight the complex interplay between miRNA regulation, metabolic dysfunction, and hypertension, opening new avenues for research into the mechanisms underlying hypertension development and potential therapeutic targets.

## Summary Table

### What Is Known About This Subject

- Hypertension is one of the leading risk factors for cardiovascular diseases and premature death globally (=107 characters)
- The limitations of existing blood pressure measurement methods make it challenging to detect early cases of hypertension (=123 characters)
- Exosomal miRNAs have been implicated in hypertension development and have potential as non-invasive disease biomarkers (=120 characters)

### What This Paper Adds

- This study provided the first comprehensive exosomal miRNAome in newly identified essential hypertensive adults (=114 characters)
- Hypertensives showed distinct extracellular vesicle morphologies and size, as well as reduced CD9 expression (=111 characters)
- An exosomal miRNA signature with BMI has been identified as a potential biomarker for the early diagnosis of hypertension (=119 characters)

## Concluding Statement

This work represents an advance in biomedical science because it identifies a unique exosomal miRNA profile for stage I hypertension when used in combination with BMI (=167 characters).

## Data Availability

The datasets presented in this study can be found in online repositories. The names of the repository/repositories and accession number(s) can be found in the article/Supplementary Material.
