# Supplementary figures and images for: Identification of an Exosomal miRNA Signature in Newly Diagnosed Essential Hypertensive Adults
Source: Br J Biomed Sci. 2025 Nov 21;82:14780. doi: 10.3389/bjbs.2025.14780 (PMC12678194; doi:10.3389/bjbs.2025.14780)

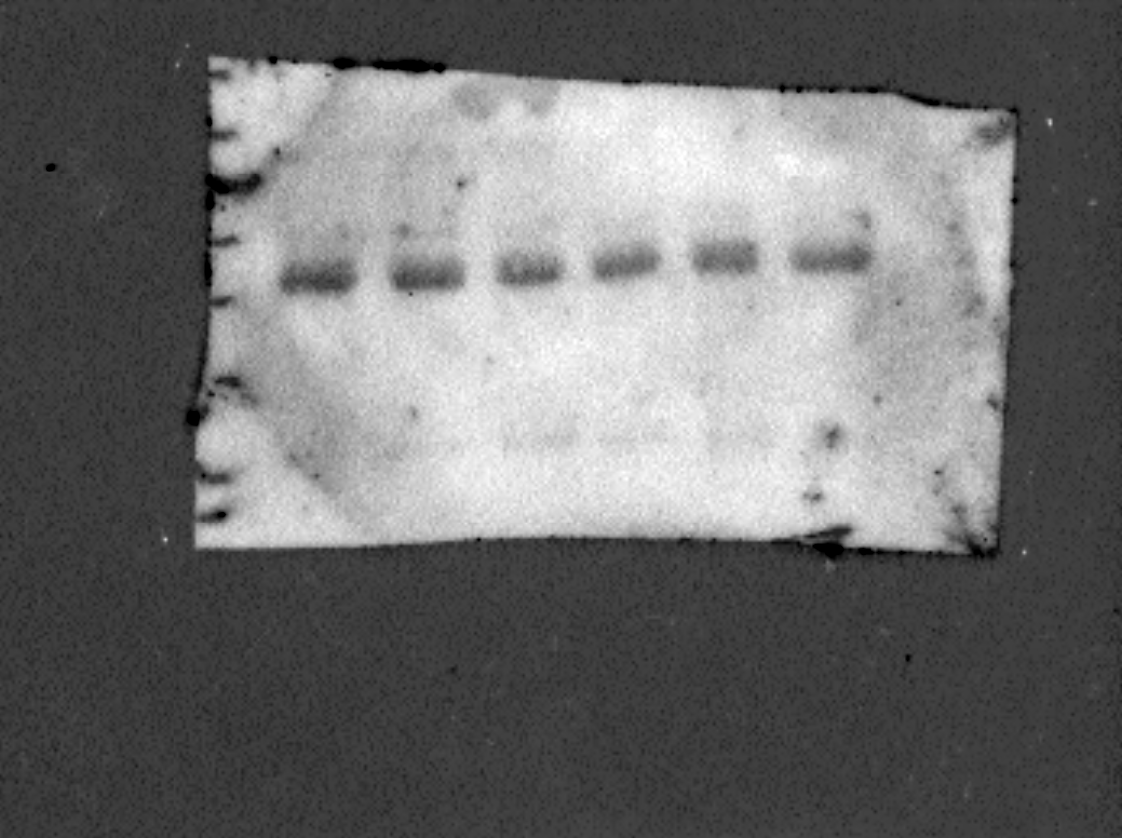

Supplement: Supplementary file 1 [file Image3.tif]

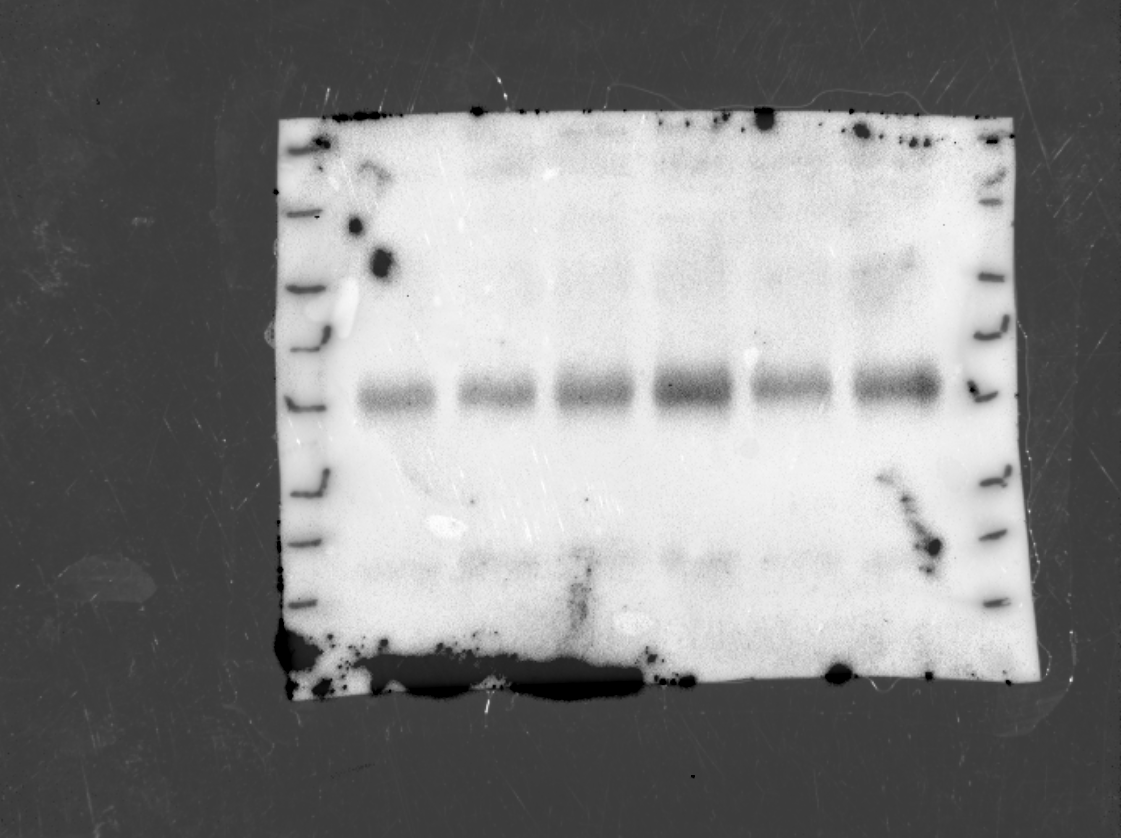

Supplement: Supplementary file 2 [file Image4.tif]

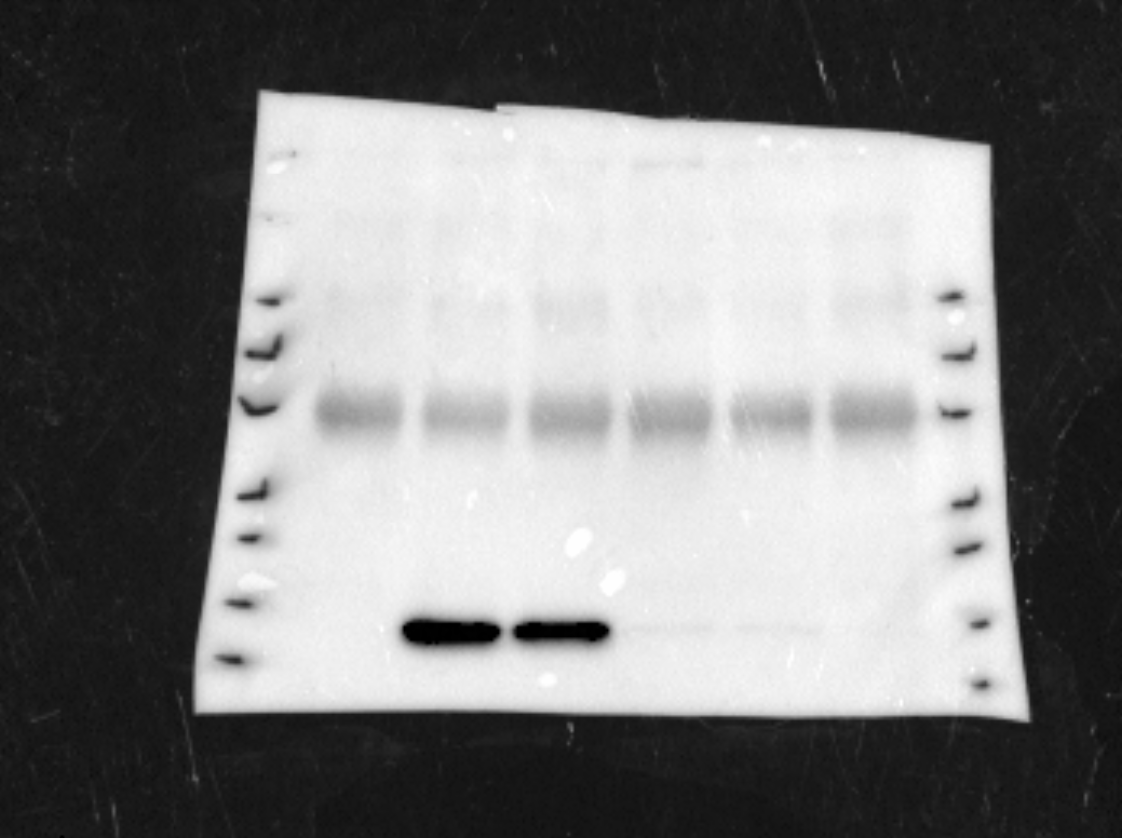

Supplement: Supplementary file 3 [file Image2.tif]

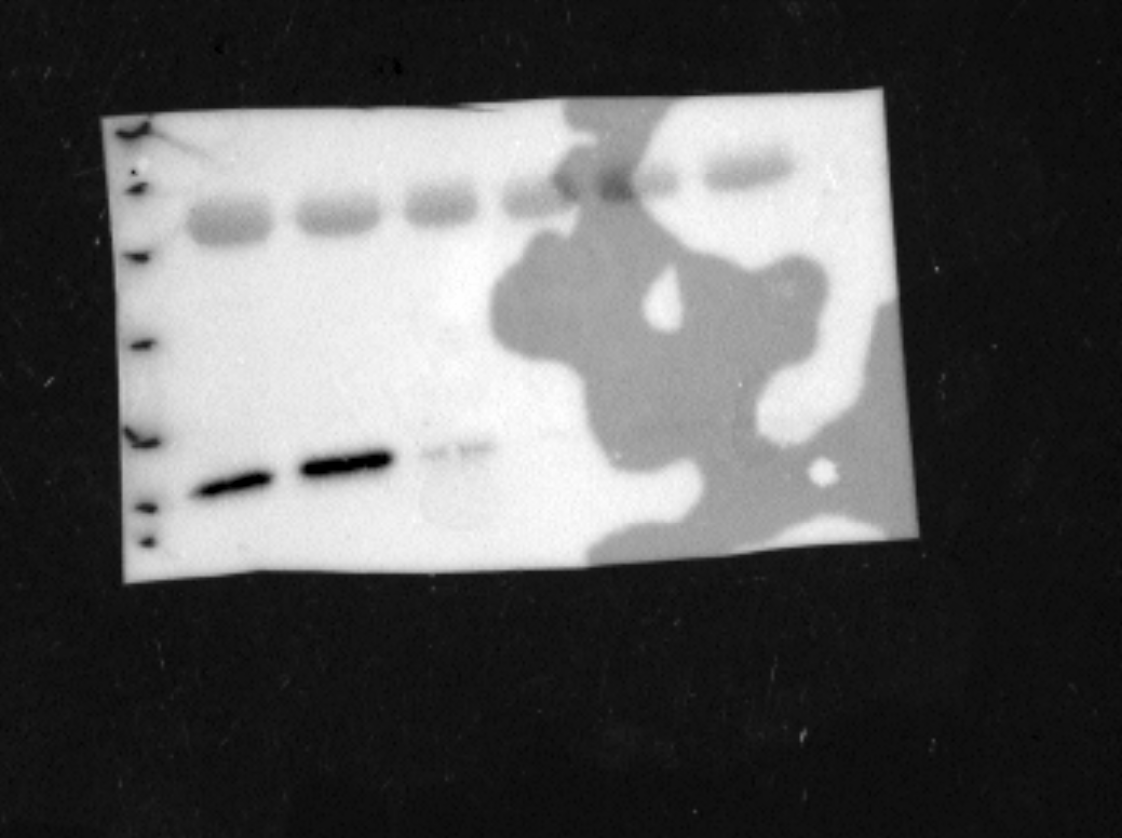

Supplement: Supplementary file 4 [file Image1.tif]
