# Supplementary material for: Identification of an Exosomal miRNA Signature in Newly Diagnosed Essential Hypertensive Adults
Source: Br J Biomed Sci. 2025 Nov 21;82:14780. doi: 10.3389/bjbs.2025.14780 (PMC12678194; doi:10.3389/bjbs.2025.14780)
Supplement: Supplementary file 5 [file DataSheet1.pdf]

## Supplementary Material 1

**Table S1. Concentration of exosomes isolated from newly diagnosed, stage I hypertensives compared to controls (n=12)**. There was no significant difference in total absolute number of particles, or when classified into three different size classes. All measurements were taken using nanoparticle tracking analysis. All measurements using nanoparticle tracking analysis were analysed from 5 x 60 s scans according to ISO guidelines for biological replicate. * Indicates *P* < 0.05 statistical difference using student's t-test.

| Size class (nm) | Number of particles (particles/ml) | | *P*-value |
|---|---|---|---|
| | Control (n=6) | Hypertensive (n=6) | |
| total | $7.1\times10^{11} \pm 2.7\times10^{11}$ | $5.4\times10^{11} \pm 4.3\times10^{10}$ | 0.325 |
| 30.5 - 150 | $6.8\times10^{11} \pm 2.6\times10^{11}$ | $5.0\times10^{11} \pm 4.2\times10^{10}$ | 0.303 |
| 150.5 - 200 | $1.8\times10^{10} \pm 1.1\times10^{10}$ | $3.1\times10^{10} \pm 1.2\times10^{9}$ | 0.293 |
| > 200 | $5.8\times10^{9} \pm 4.7\times10^{9}$ | $1.2\times10^{10} \pm 6.3\times10^{9}$ | 0.541 |

**Table S2. Participants selected for next generation sequencing (n=12)**. Systolic blood pressure, diastolic blood pressure and body mass index were significantly increased in hypertensives than controls. Data is expressed as mean ± SD or n (%). * Indicates *P* < 0.05 using Chi-square test or Mann-Whitney *U* test as appropriate.

| **Variables** | **Control (n = 6)** | **Hypertensive (n = 6)** | ***P*-value** |
|---|---|---|---|
| **Age (years)** | 39.7 ± 9.2 | 43.5 ± 5.6 | |
| **Sex** | | | |
| Male | 4 (66.7) | 4 (66.7) | 0.819[a] |
| Female | 2 (33.3) | 2 (33.3) | |
| **Race** | | | |
| Malay | 2 (33.3) | 2 (33.3) | 0.819[a] |
| Chinese | 4 (66.7) | 4 (66.7) | |
| **Family history of hypertension** | | | |
| no | 1 (16.7) | 1 (16.7) | 0.727[a] |
| yes | 5 (83.3) | 5 (83.3) | |
| **SBP (mmHg)** | 110.8 ± 12.7 | 147.3 ± 9.6 | < 0.004[b] * |
| **DBP (mmHg)** | 73.3 ± 10.3 | 94.5 ± 7.1 | < 0.004[b] * |
| **BMI (kg/cm$^2$)** | 23.4 ± 1.4 | 31.5 ± 2.4 | 0.004[b]* |
| **WC** | | | |
| normal | 3 (50.0) | 0 (0.0) | 0.087[a] |
| abdominal obesity | 3 (50.0) | 6 (100.0) | |
| **TC (mg/dL)** | 5.7 ± 0.6 | 5.20 ± 1.0 | 0.339[b] |
| **HDL (mg/dL)** | 1.4 ± 0.4 | 1.2 ± 0.3 | 0.177[b] |
| **LDL (mg/dL)** | 3.4 ± 0.3 | 3.6 ± 0.7 | 1.000[b] |
| **TG (mg/dL)** | 2.2 ± 2.3 | 1.0 ± 1.2 | 0.662[b] |
| **TC/HDL ratio** | 4.4 ± 2.0 | 4.4 ± 1.1 | 0.247[b] |
| **Plasma glucose** | 4.6 ± 0.9 | 5.2 ± 1.0 | 0.310 [b] |

[a]Chi-square test
[b]Mann-Whitney *U* test
SBP, systolic blood pressure; DBP, diastolic blood pressure; BMI, body mass index; WC, waist circumference; TC, total cholesterol; HDL, high density lipoprotein; LDL, low density lipoprotein; TG, triglyceride

**Table S3. Overall mapping statistics of retained reads to mature miRNA in miRbase (n=12).** Total reads mapped refer to the sum of reads annotated with miRbase and ambiguously annotated reads. Reads annotated with miRbase were used for differential expression analysis, whereas ambiguously annotated reads were discarded. Data is expressed as the number of reads mapped against retained reads post-trimming.

| Sample | Input reads | Total reads mapped | | Annotated with miRbase | | Ambiguously annotated | |
|---|---|---|---|---|---|---|---|
| | | Number of reads | % | Number of reads | % | Number of reads | % |
| C09 | 6,627,258 | 5,131,046 | 77.4 | 3,211,010 | 48.5 | 1,920,036 | 29.0 |
| C10 | 4,530,656 | 2,115,860 | 46.7 | 1,394,146 | 30.8 | 721,714 | 15.9 |
| C12 | 3,510,849 | 3,000,402 | 85.5 | 1,773,808 | 50.5 | 1,226,594 | 34.9 |
| C13 | 2,985,083 | 2,007,127 | 67.2 | 1,207,942 | 40.5 | 799,185 | 26.8 |
| C15 | 2,898,795 | 1,249,619 | 43.1 | 791,330 | 27.3 | 458,289 | 15.8 |
| C16 | 1,474,238 | 494,760 | 33.6 | 322,494 | 21.9 | 172,266 | 11.7 |
| H10 | 4,071,497 | 1,257,307 | 30.9 | 819,607 | 20.1 | 437,700 | 10.8 |
| H11 | 4,064,291 | 2,145,598 | 52.8 | 1,345,378 | 33.1 | 800,220 | 19.7 |
| H12 | 4,507,111 | 1,447,763 | 32.1 | 918,382 | 20.4 | 529,381 | 11.8 |
| H13 | 5,365,638 | 1,304,469 | 24.3 | 838,736 | 15.6 | 465,733 | 8.7 |
| H15 | 2,565,270 | 1,718,242 | 67.0 | 1,051,320 | 41.0 | 666,922 | 26.0 |
| H16 | 3,715,667 | 2,132,940 | 57.4 | 1,297,144 | 34.9 | 835,796 | 22.5 |
| **mean** | 3,859,696 | 2,000,428 | 51.5 | 1,247,608 | 32.1 | 752,820 | 19.5 |
| **SD** | 1,351,093 | 1,171,833 | 19.8 | 721,627 | 11.5 | 453,463.3 | 8.4 |

**Table S4. Cut-off points for classification of miRNA expression from receiver's operating characteristic (ROC) curve analysis.** Youden's index was calculated and used as cut-off points for statistically significant ($P < 0.05$) ROC models based on the validation cohort (n=35). Median was used as a cut-off point for statistically insignificant ($P > 0.05$) ROC models.

| miRNA | AUC | 95% CI | *P*-value | Cut-off | Se (%) | Sp (%) |
|---|---|---|---|---|---|---|
| | | | *Downregulated miRNA* | | | |
| hsa-miR-1-3p | 0.688 | 0.510-0.865 | 0.039* | 2.076[a] | 35.7 | 88.9 |
| hsa-miR-184 | 0.733 | 0.553-0.914 | 0.011* | 0.822[a] | 42.9 | 88.9 |
| hsa-miR-432-5p | 0.701 | 0.509-0.893 | 0.04* | 0.340[a] | 85.7 | 77.8 |
| hsa-miR-206 | 0.587 | 0.371-0.804 | 0.429 | 1.738[a] | 92.9 | 44.4 |
| hsa-miR-199a-3p | 0.496 | 0.286-0.705 | 0.968 | 0.940[b] | - | - |
| hsa-let-7c-5p | 0.526 | 0.320-732 | 0.805 | 0.101[b] | - | - |
| | | | *Upregulated miRNA* | | | |
| hsa-miR-1246 | 0.695 | 0.512-0.878 | 0.037* | -0.454[a] | 82.4 | 56.2 |
| hsa-miR-4508 | 0.422 | 0.214-0.630 | 0.464 | -0.351[b] | - | - |

[a]Youden's index
[b]Median
ROC, receiver's operating characteristic; AUC, area under the curve; CI, confidence interval; Se, sensitivity; Sp, specificity

**Figure S1. Mapping statistics of retained reads (n=12).** After adapter trimming, the proportion of retained reads from controls (**A**) and hypertensives (**B**) to mature miRNA in miRbase were evaluated.

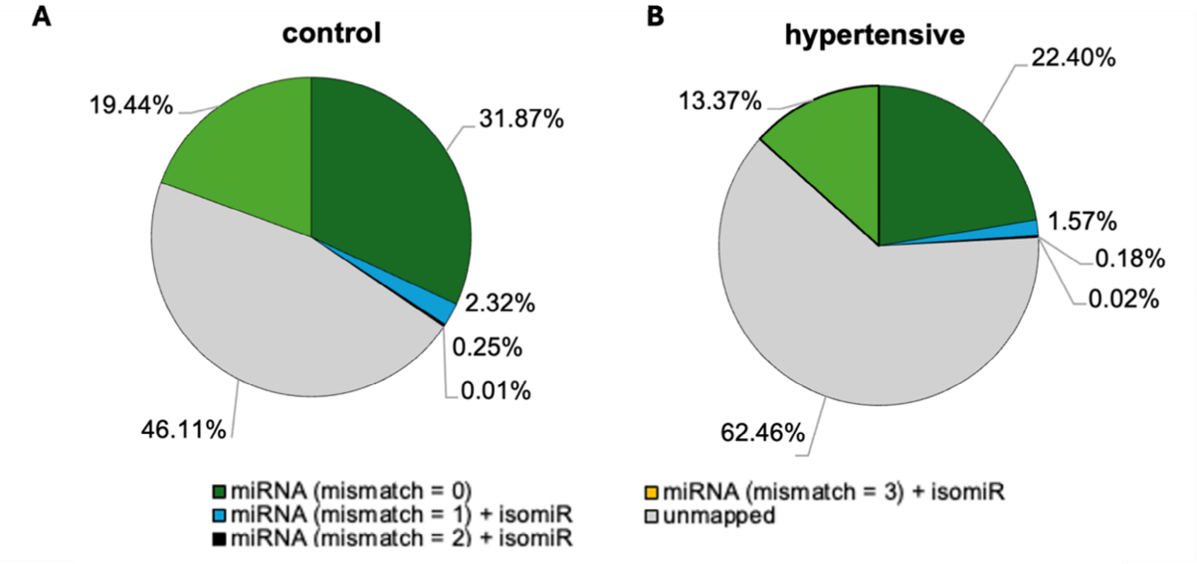

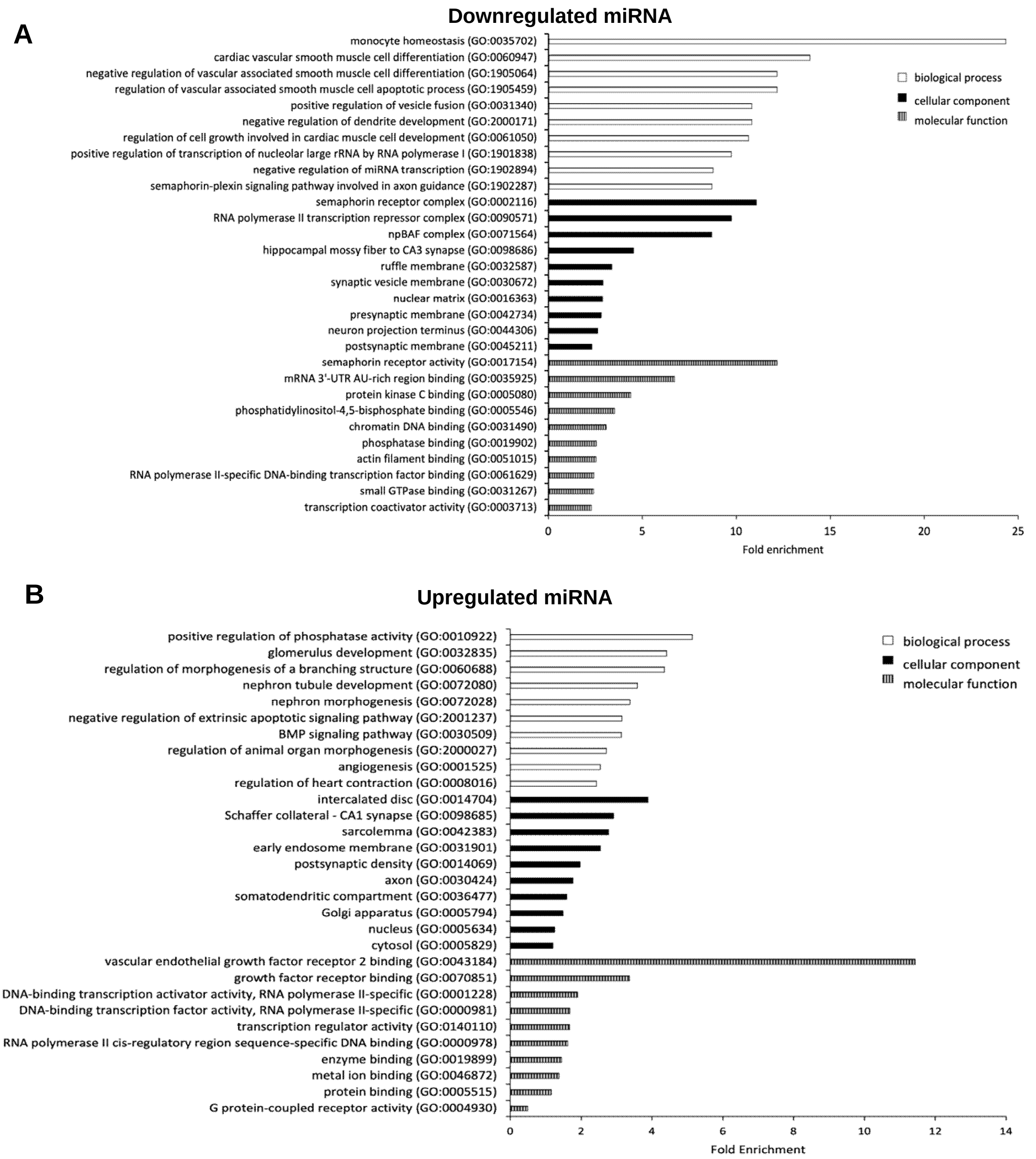


**Figure S2. Gene ontology enrichment analysis of target genes from the hypertension exosomal miRNA signature.** Top ten most enriched gene ontology (GO) terms for each category were chosen for the predicted target genes from hsa-miR-1-3p, hsa-miR-184 and hsa-miR-432-5p (**A**), and from hsa-miR-1246 (**B**). GO terms were considered enriched when FDR < 0.05. GO, gene ontology, FDR, false discovery rate.

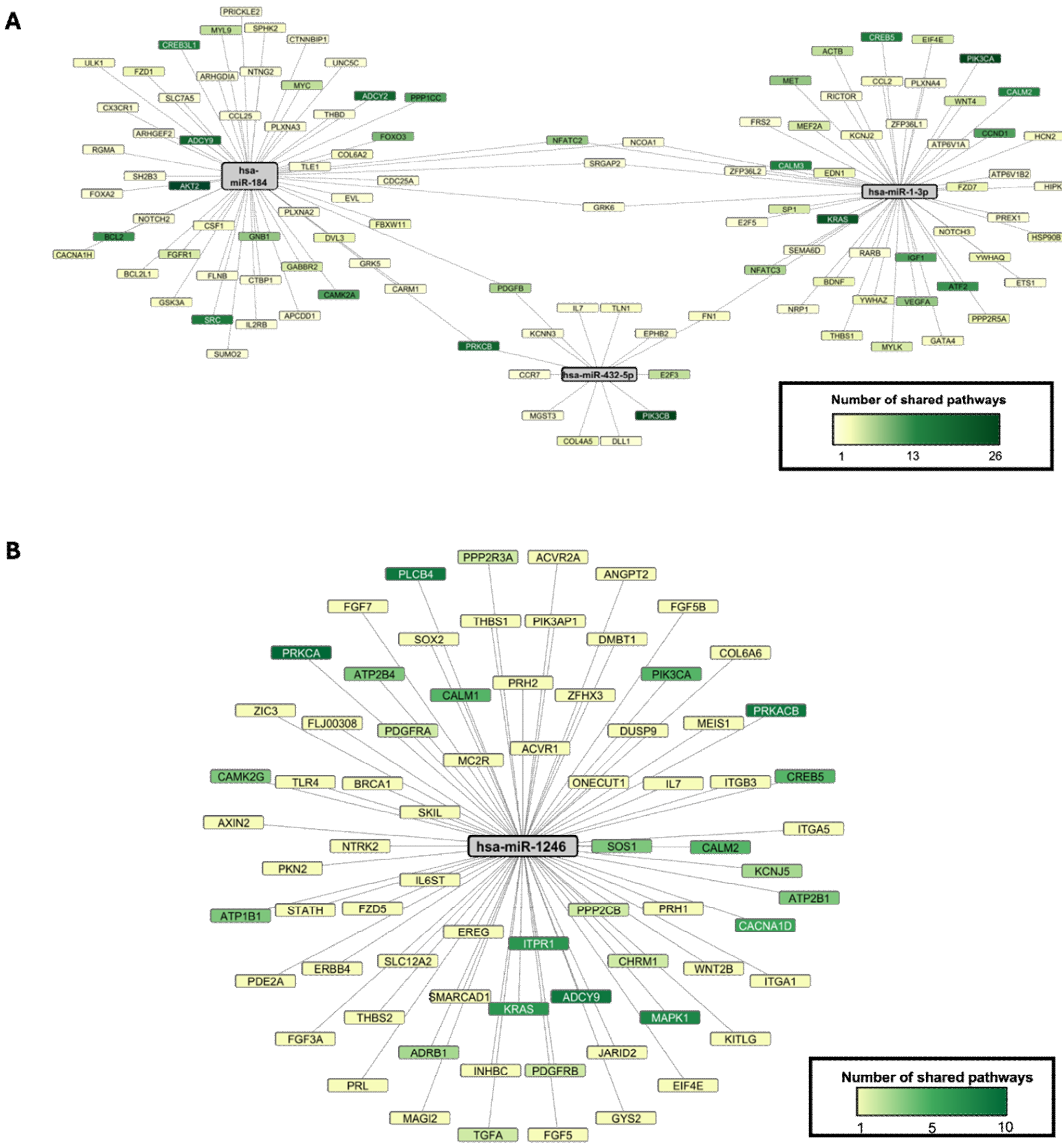


**Figure S3. miRNA-mRNA network interaction network of downregulated and upregulated miRNAs.** miRNA targets were selected from the KEGG enriched pathways when FDR < 0.05. Target genes associated with higher number of shared pathways were shaded with a darker shade of green. (**A**) From the miRNA-target gene network of has-miR-184, hsa-miR-1-3p, and hsa-miR-432-5p, *PIK3CB, PRKCB, PIK3CA, ADCY2, CALM2, CREB3L1, ADCY9, AKT2, SRC, KRAS,* and *CREB5* were overrepresented. (**B**) In the *hsa-miR-1246*-target gene network, genes encoding for *PIK3CA, ATP2B4, ATP2B1, KRAS, CREB5, PLCB4, MAPK1, CACNA1D, ITPR1, CALM1, CAMK2G, CALM2, PRKACB,* and *ATP1B1* were overrepresented.

**Table S5. Hallmarks associated with circulating proteins between hypertensives and controls (n=6).** Enrichment and its associated statistics was conducted using GSEA analysis.

| GSEA Hallmark | Enrichment | *P*-value | FDR-value |
|---|---|---|---|
| | *Downregulated pathways* | | |
| Heme metabolism | -2.279 | 0.000 | 0.000 |
| PI3K/AKT/MTOR signalling | -2.030 | 0.000 | 0.001 |
| MYC targets | -1.854 | 0.000 | 0.005 |
| Fatty acid metabolism | -1.828 | 0.000 | 0.006 |
| Oxidative phosphorylation | -1.745 | 0.000 | 0.011 |
| ROS pathway | -1.637 | 0.012 | 0.031 |
| MTORC1 signalling | -1.634 | 0.001 | 0.028 |
| | *Upregulated pathways* | | |
| Pancreas beta cells | 1.704 | 0.010 | 0.016 |

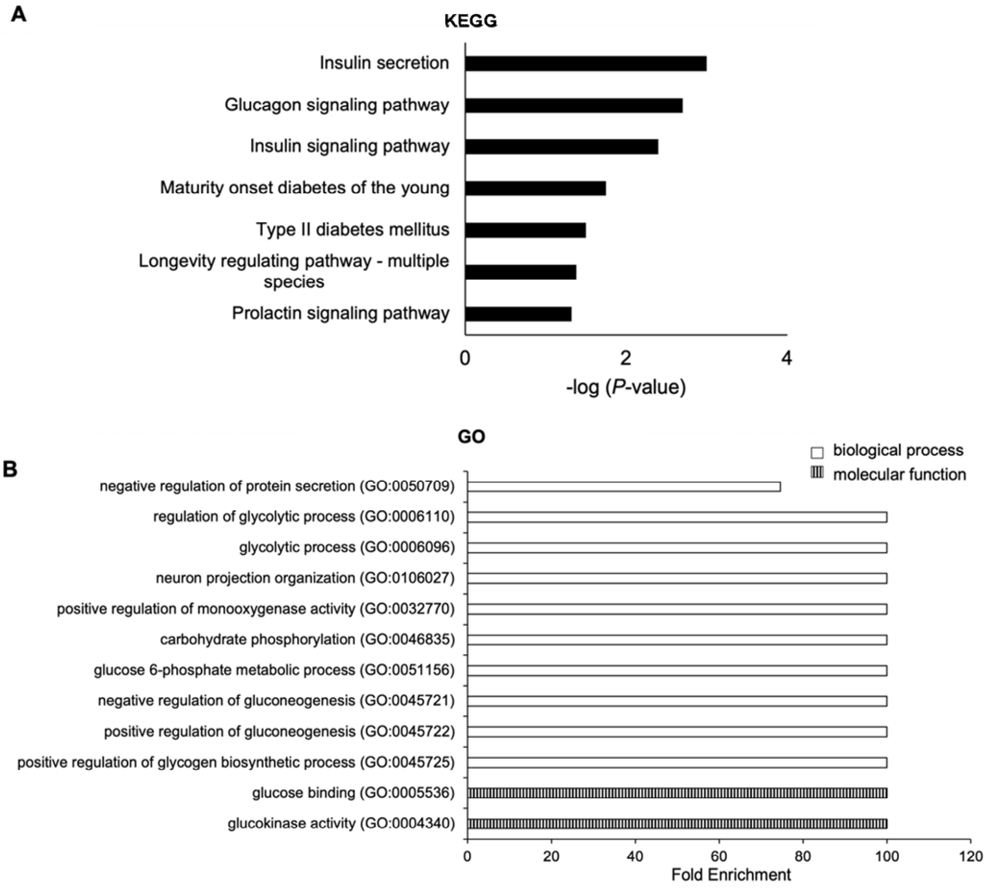


**Figure S4. Top ten enriched KEGG pathways and GO of leading-edge genes from upregulated hallmarks associated with circulating proteins of newly identified hypertensives. (A)** Systemically increased insulin signalling and **(B)** processes associated with energy metabolism were observed in newly diagnosed hypertensives. *P*-value was generated using standard gene ontology and KEGG analysis pipeline using the DAVID bioinformatics tool. *P*-value was used as the cut-off point for identifying enriched KEGG pathways associated upregulated hallmarks as no statistically significant pathways were identified if FDR-value was used.
